# Supplementary material for: Remodeling of the postsynaptic proteome in male mice and marmosets during synapse development
Source: Nat Commun. 2024 Mar 28;15:2496. doi: 10.1038/s41467-024-46529-9 (PMC10979008; doi:10.1038/s41467-024-46529-9)
Supplement: Supplementary file 1 — Supplementary Information [file 41467_2024_46529_MOESM1_ESM.pdf]

**Supplementary Information**

# Remodeling of the postsynaptic proteome in male mice and marmosets during synapse development

Takeshi Kaizuka[1,2*], Takehiro Suzuki[3*], Noriyuki Kishi[1], Kota Tamada[1,2], Manfred W. Kilimann[4], Takehiko Ueyama[5], Masahiko Watanabe[6], Tomomi Shimogori[1], Hideyuki Okano[1,7], Naoshi Dohmae[3], Toru Takumi[1,2,8,9]

1 RIKEN Brain Science Institute, Wako, Saitama 351-0198, Japan.
2 Department Physiology and Cell Biology, Kobe University School of Medicine, Chuo, Kobe 650-0117, Japan.
3 Biomolecular Characterization Unit, RIKEN Center for Sustainable Resource Science, Wako, Saitama 351-0198, Japan.
4 Max Planck Institute for Experimental Medicine, Göttingen, 37075, Germany.
5 Laboratory of Molecular Pharmacology, Biosignal Research Center, Kobe University, Nada, Kobe 657-8501, Japan
6 Department of Anatomy, Faculty of Medicine, Hokkaido University, Kita, Sapporo 060-8638, Japan
7 Department of Physiology, Keio University School of Medicine, Shinjuku, Tokyo 160-8585, Japan.
8 RIKEN Center for Biosystems Dynamics Research, Chuo, Kobe 650-0047, Japan
9 Lead Contact
*These authors equally contributed to the work

Correspondence should be addressed to Toru Takumi takumit@med.kobe-u.ac.jp

**This PDF file includes:**
Figs. S1 to S18
Supplementary References

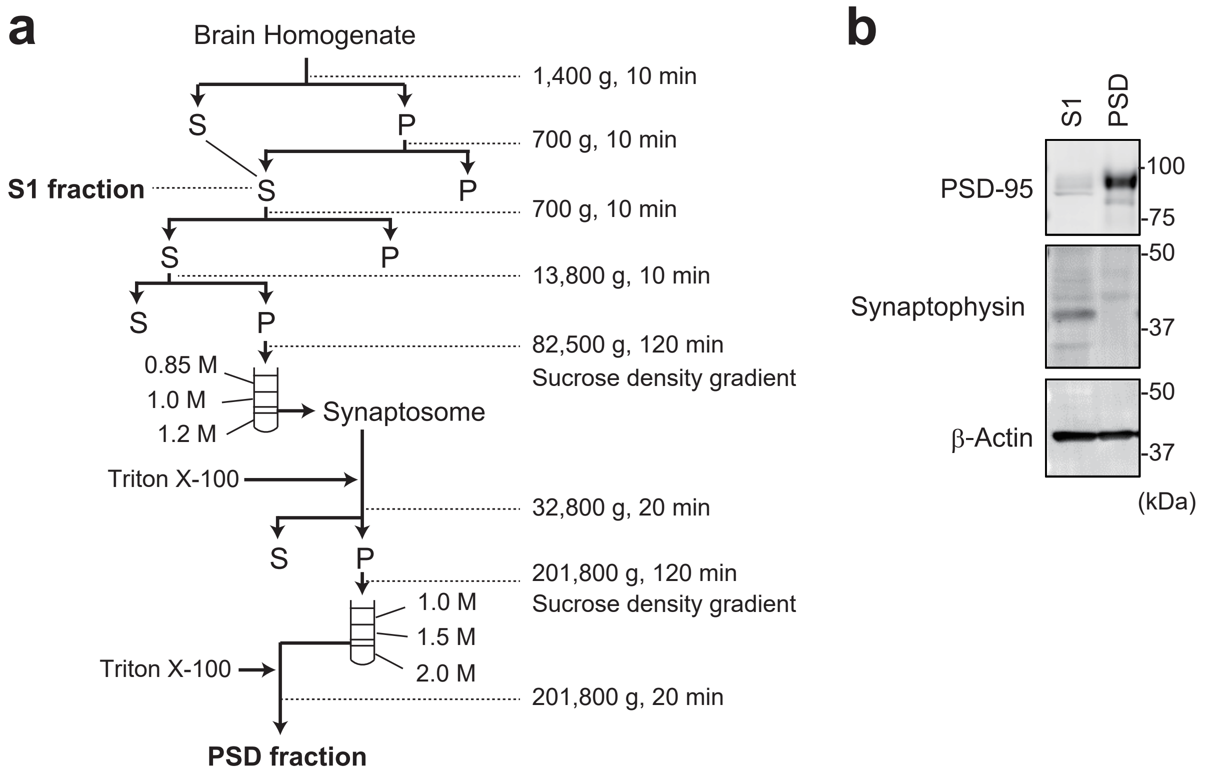


**Supplementary Fig. 1. Biochemical purification and analysis of the postsynaptic density (PSD) from mouse brain** **(a)** Procedure of PSD purification by differential centrifugation and sucrose density gradient centrifugation (Modified from Ref 1. S and P indicate supernatant and pellet, respectively. **(b)** Confirmation of PSD purification. PSD fraction was prepared from adult (12-week-old) ICR mouse brains. S1 and PSD fractions are shown in (a) were subjected to SDS-PAGE followed by Western blotting. Source data are provided as a Source Data file.

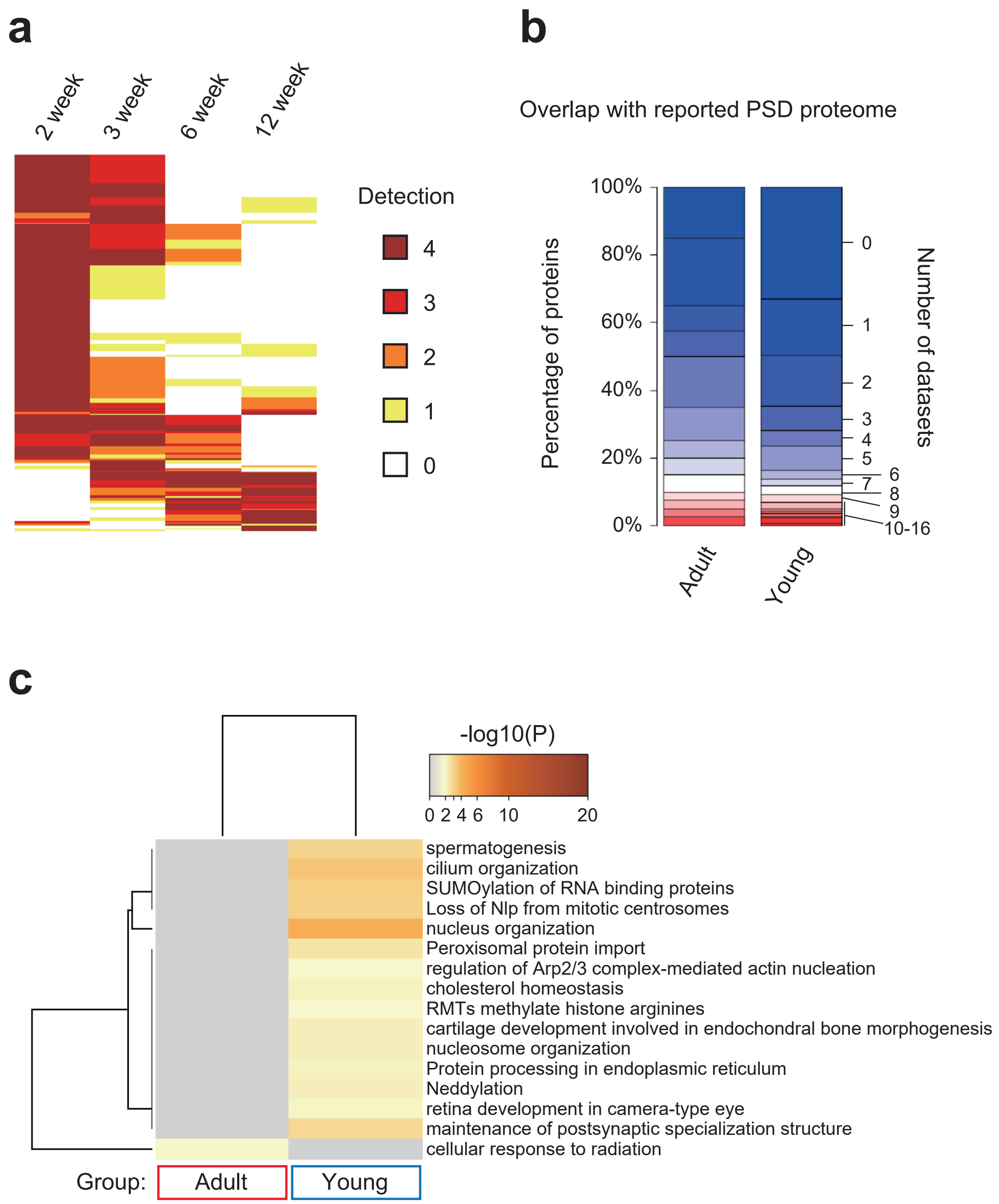


**Supplementary Fig. 2. Proteins detected in specific stages of mouse PSD**

**(a)** Number of detection of proteins with high confidence. 270 proteins that were detected in 4 of 4 replicates and 0 of 4 replicates in at least one age in mouse PSD proteome dataset (Supplementary Data 1) are plotted. **(b)** Overlap between the proteins in each cluster and 16 previously published PSD proteome datasets. Adult: 40 proteins specifically detected in PSD of adult mice (6~12 week old), Young: 221 proteins specifically detected in PSD of young age mice (2~3 week old). **(c)** Statistically enriched Gene Ontology terms and pathway terms in each cluster identified by Metascape. The top 20 terms are displayed as a hierarchically clustered heatmap. The heatmap cells are colored by their P-values; grey cells indicate the lack of enrichment for that term in the corresponding protein list. Source data are provided as a Source Data file.

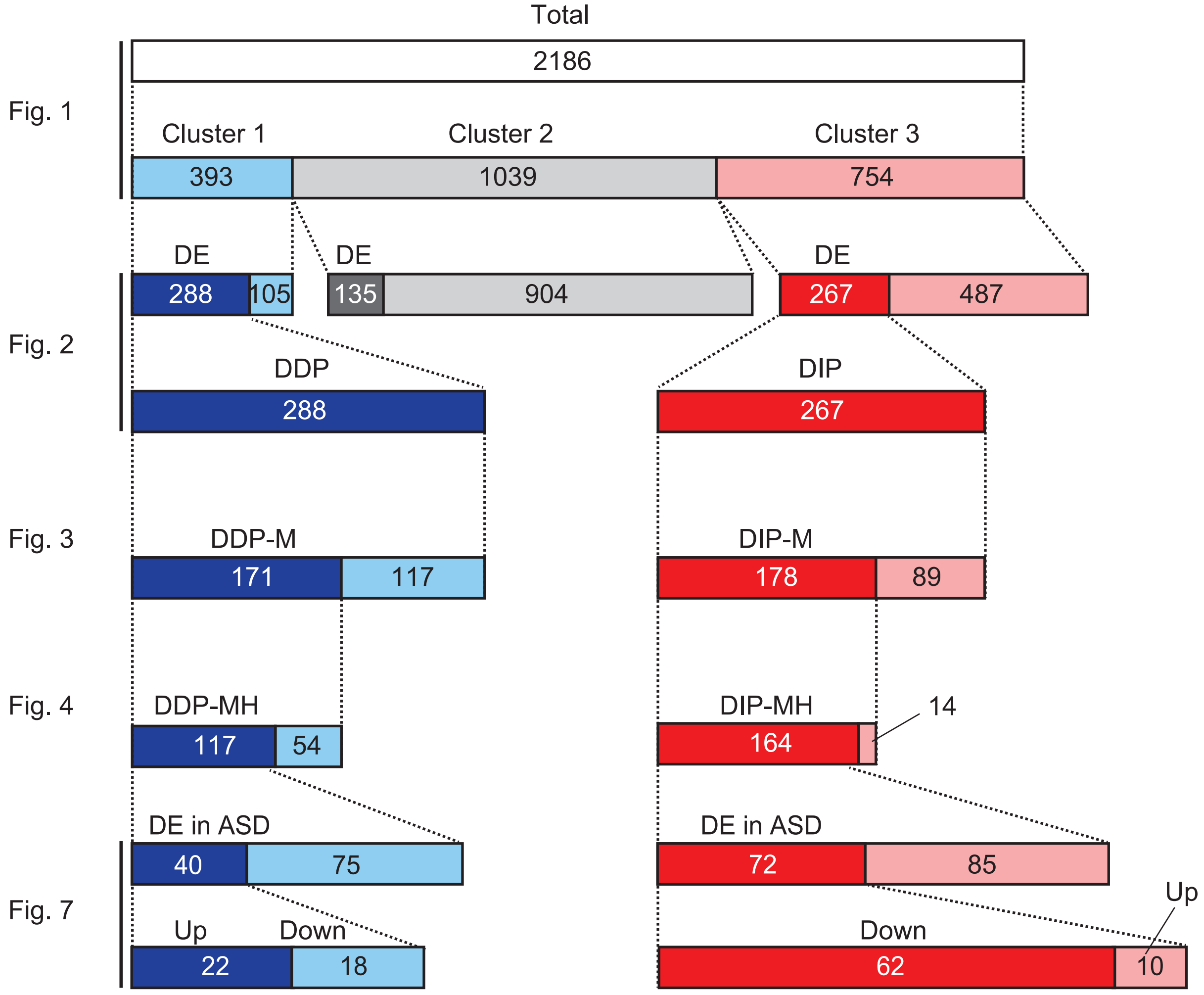


**Supplementary Fig. 3. Overall workflow of protein classification**

In this study, 2,186 PSD proteins were analyzed (Fig. 1a, 1b). The proteins were classified into 3 clusters using k-means clustering (Fig. 1c). Differentially expressed (DE) proteins were identified using criteria: max fold change > 1.5, and P-value < 0.05. DE proteins in Cluster 1 were defined as DDP proteins, whereas those in Cluster 3 were defined as DIP proteins (Fig. 2a, 2b). Among DDP and DIP proteins, those which showed a positive correlation with gene expression were termed as DDP-M and DIP-M, respectively, according to the correlation between the change of protein abundance and that of mRNA abundance in mice (Supplementary Fig. 5b, 5c). Among the proteins in DDP-M and DIP-M, those that showed a positive correlation with gene expression in the human brain were termed as DDP-MH and DIP-MH based on the correlation with the human transcriptome (Fig. 4a). Genes encoding DDP-MH and DIP-MH proteins, 42 and 72, respectively, were reported to be differentially expressed in patients with ASD (Fig. 7a).

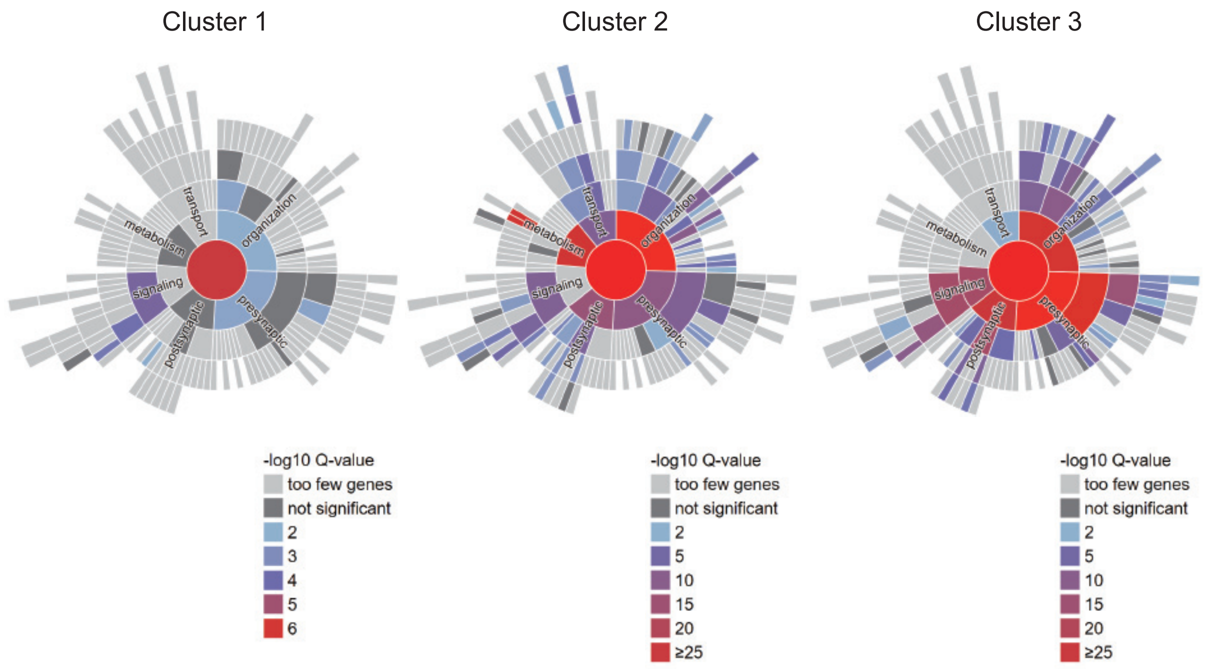


**Supplementary Fig. 4. Analysis of postsynaptic protein clusters with SynGO**

Statistically enriched Gene Ontology terms and pathway terms in each cluster identified by SynGO.

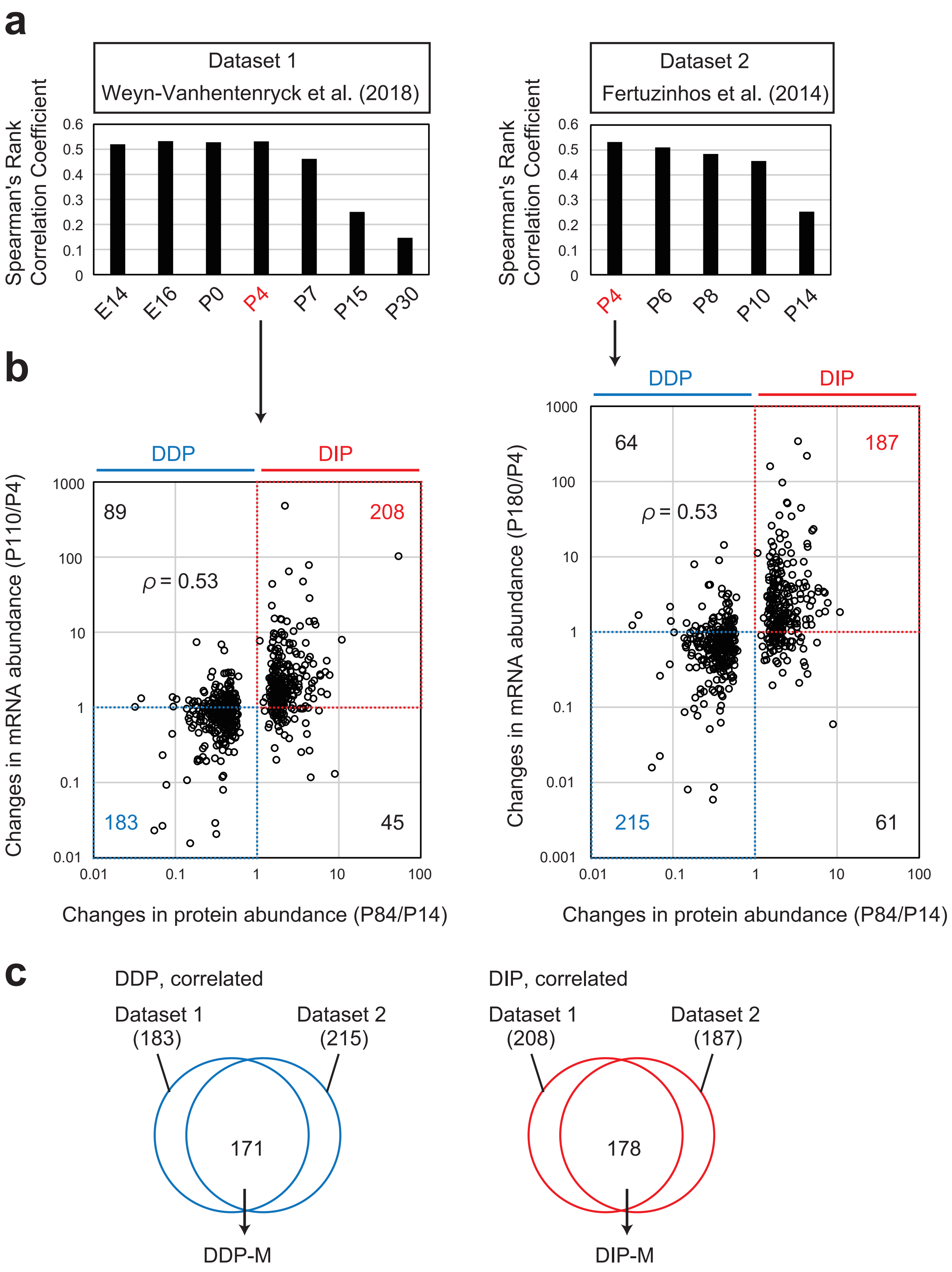


**Supplementary Fig. 5. Correlation between the alteration of PSD protein abundance and mRNA abundance in mice during postnatal development**

**(a)** Spearman's rank correlation coefficient of changes in relative PSD protein abundance and mRNA abundance. For PSD protein, changes from 2-week-old to 12-week-old were used. For mRNA abundance, changes from indicated age to P110 or P180 in Dataset 1 (Ref 2) and Dataset 2 (Ref 3) are used, respectively. **(b)** Fold change of the abundance of DE PSD proteins in the developing mouse brain (12-week old vs. 2-week old) was plotted against mRNA abundance in the developmental mouse cortex. The number of proteins in indicated group and Spearman's rank correlation coefficient are shown. **(c)** Extraction of proteins whose abundance is correlated with mRNA abundance. DDP-M is a subgroup of DDP whose mRNA is decreased after P4 in both transcriptome datasets. DIP-M is a subgroup of DIP as well. Source data are provided as a Source Data file.

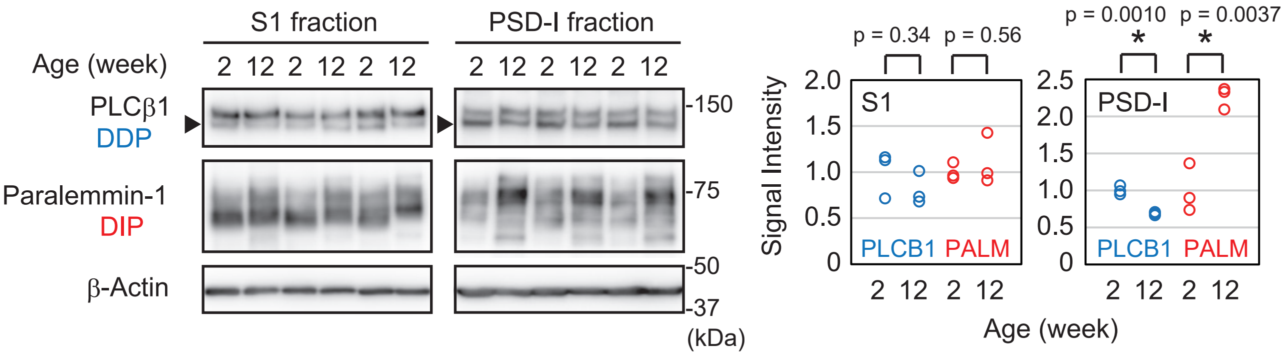


**Supplementary Fig. 6. Developmental alteration of PSD localization of DDP or DIP protein which does not show a positive correlation with transcriptional changes.**

20 μg of proteins obtained from the indicated fraction was loaded and analyzed by immunoblotting using antibodies against PLCβ1 (PLCB1) or Paralemmin-1 (PALM). Samples were independently prepared from three biological replicates. Quantification of band intensity is shown on the right. Two isoforms of PLCβ1 were detected and the bottom band (shown as an arrowhead) is evaluated because it is thought to be the isoform we detected in the MS dataset. All immunopositive bands of Paralemmin-1 were pooled for quantification as Paralemmin-1 is known to give multiple bands on Western blots due to differential splicing as well as phosphorylation (Ref 4).

*P < 0.01 (Two-sided unpaired Student's t-test) Source data are provided as a Source Data file.

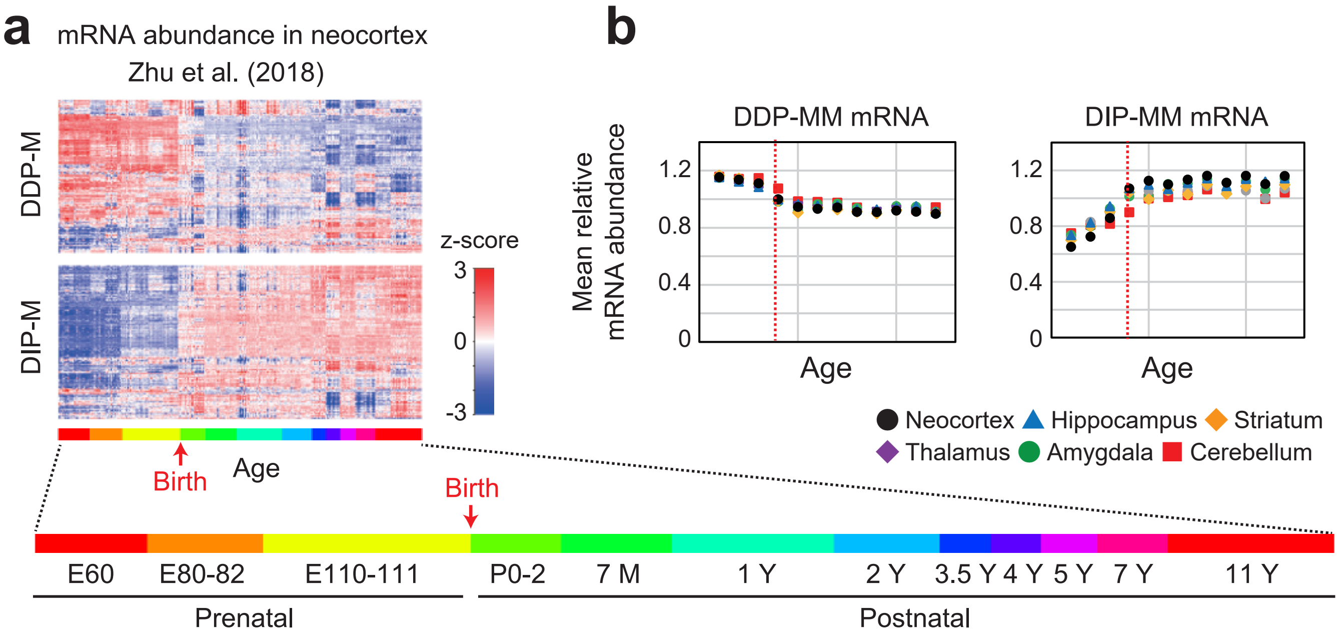


**Supplementary Fig. 7. Transcriptional profiles of PSD genes in developing macaque brain**

**(a)** Heatmap of the relative abundance of mRNAs that encode DDP-M and DIP-M PSD proteins in cortical regions of the developing macaque brain (Ref 5). **(b)** The plot of mean relative abundance of mRNAs that encode PSD proteins in the groups DDP-MM (left) or DIP-MM (right) (subgroups of DDP-M or DIP-M whose mRNA was increased or decreased in macaque cortex after birth, respectively). The 12-time points of age are described in (a). Red dotted lines indicate birth. Source data are provided as a Source Data file.

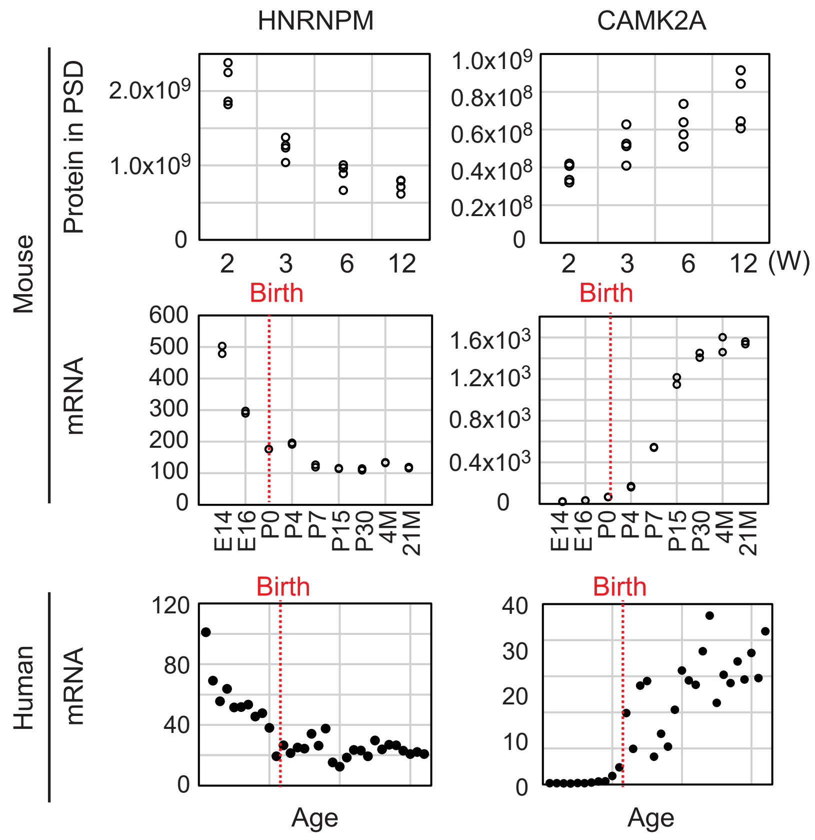


**Supplementary Fig. 8. Representative examples of PSD protein abundance in mice and gene expressionin mouse and human**

Representative examples of proteins in DDP-MH (left, HNRMPM) and DIP-MH (right, CAMKK2A). (top) Protein abundance in the PSD obtained from mouse brains (Supplementary Data 2). (middle) mRNA abundance in the mouse cortex (Ref 2). (bottom) mRNA abundance in the human brain (Ref 6). Source data are provided as a Source Data file.

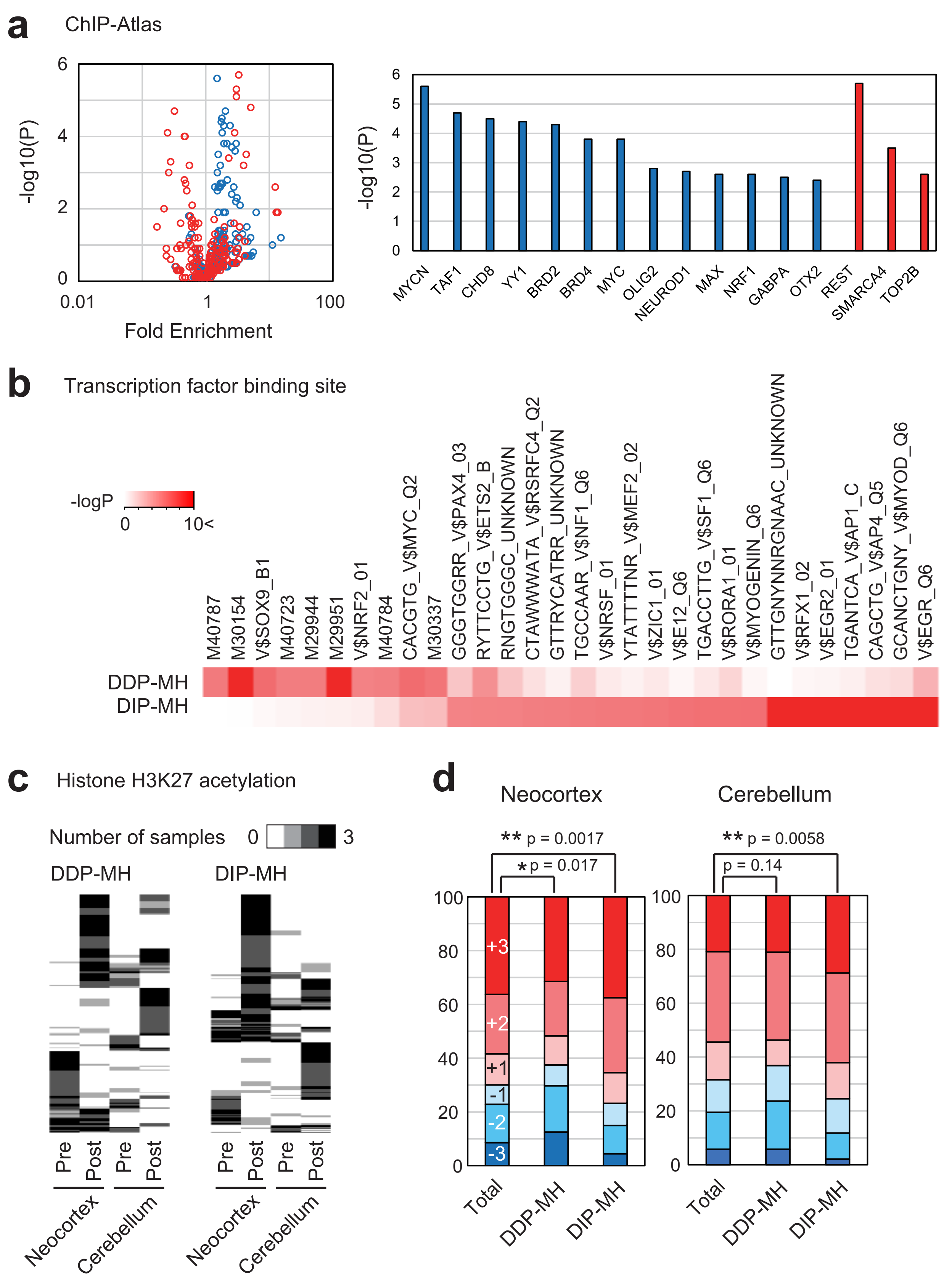


**Supplementary Fig. 9. Upstream transcription factors of genes encoding differentially expressed proteins on PSD**
**(a)** Proteins reported binding to DDP-MH genes and DIP-MH genes in human neuronal cells (antigen class: TFs and others). The enrichment was analyzed with ChIP-Atlas (Ref 7). **(b)** Enrichment of transcription factor binding sites analyzed with ToppCluster. **(c and d)** Histone H3K27 acetylation of DDP-MH and DIP-MH genes (Ref 6). **(c)** Differentially acetylated peaks around DDP-MH genes and DIP-MH genes. The number of samples in which acetylation is detected was described. Pre: prenatal (17, 19, and 21 weeks after conception), Post: postnatal (23, 30, and 37 years old). **(d)** Alteration of acetylation level between prenatal and postnatal period in Neocortex and Cerebellum. *p<0.05, **p<0.01 (Fisher's exact test). Source data are provided as a Source Data file.

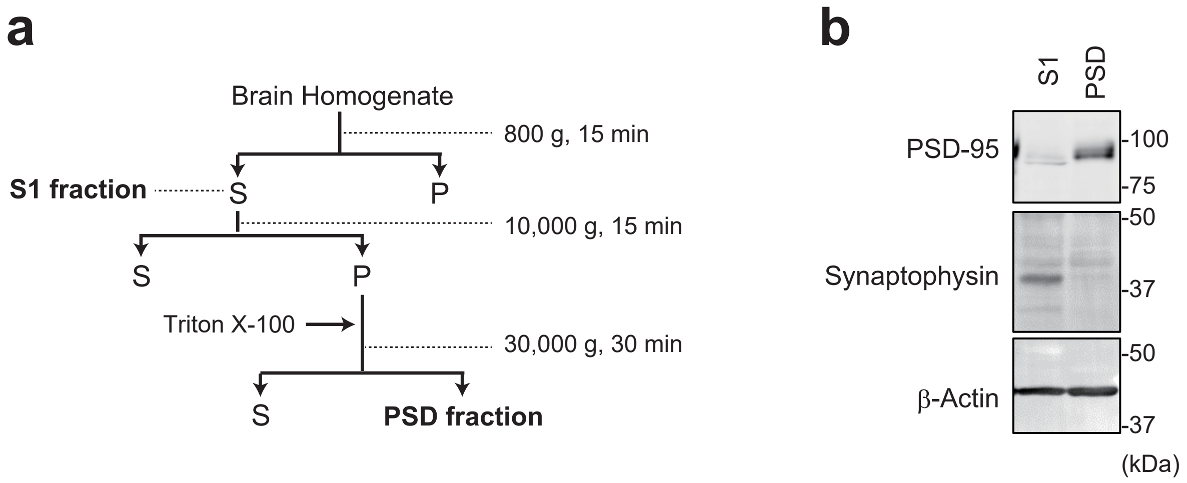


**Supplementary Fig. 10. Biochemical purification of the PSD using the 3-step method**

**(a)** Procedure of PSD purification by 3-step method (Modified from Ref 8. S and P indicate supernatant and pellet, respectively. **(b)** Confirmation of PSD purification. PSD fraction was prepared from adult (12-week-old) ICR mouse brains. S1 and PSD fractions are shown in (a) were subjected to SDS-PAGE followed by Western blotting. Source data are provided as a Source Data file.

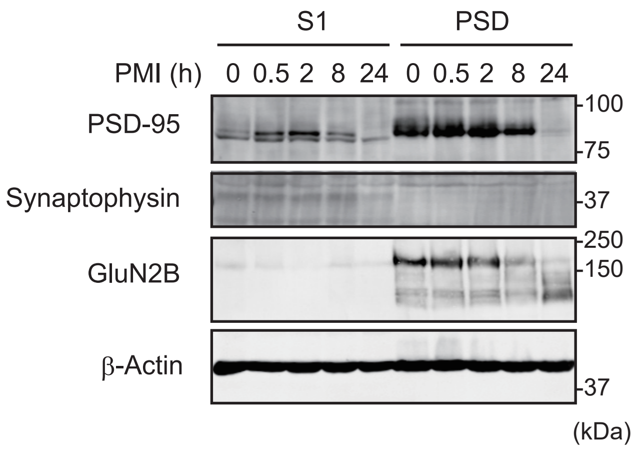


**Supplementary Fig. 11. Degradation of PSD proteins during postmortem interval**

Wild-type ICR mice were sacrificed. After the indicated duration at room temperature, whole brains were sampled. After purification of PSD using a 3-step method, proteins were analyzed with Western blotting. Source data are provided as a Source Data file.

**a** List of animals

| Age | DOB | Sacrificed | Postnatal Days | Weight (g) |
|---|---|---|---|---|
| 0 M | 2022/8/15 | 2022/8/15 | 0 | 28.7 |
| 0 M | 2022/9/25 | 2022/9/25 | 0 | 29.9 |
| 2 M | 2018/9/27 | 2018/11/27 | 61 | 106 |
| 2 M | 2022/9/9 | 2022/11/9 | 61 | 98.3 |
| 3 M | 2018/8/2 | 2018/11/1 | 91 | 146 |
| 3 M | 2022/9/9 | 2022/12/9 | 91 | 124 |
| 6 M | 2018/6/10 | 2018/12/12 | 185 | 216 |
| 6 M | 2022/1/27 | 2022/8/17 | 202 | 262 |
| 24 M | 2017/2/21 | 2019/2/21 | 730 | 489 |
| 24 M | 2020/10/13 | 2022/10/14 | 731 | 366 |

**b** Marmoset brain

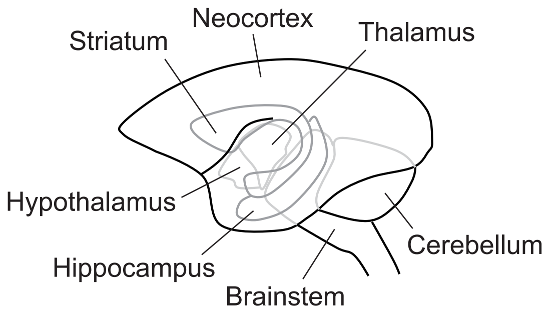


**c**

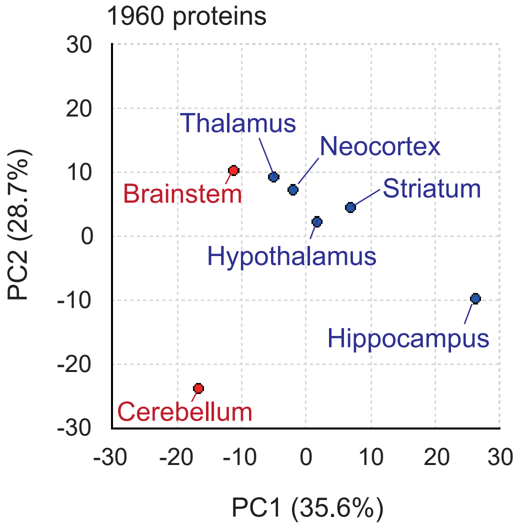


**Supplementary Fig. 12. PSD proteome analysis of common marmoset brain**

**(a)** List of the marmosets used in the present study. DOB: date of birth. **(b)** Seven brain regions of marmoset. **(c)** PSD samples prepared from individual brain regions of an adult (24-month-old) marmoset were subjected to LC-MS/MS for label-free quantification. PCA was performed using the mean relative abundance values of 1,960 proteins. Blue and red points indicate forebrain and hindbrain regions, respectively. Source data are provided as a Source Data file.

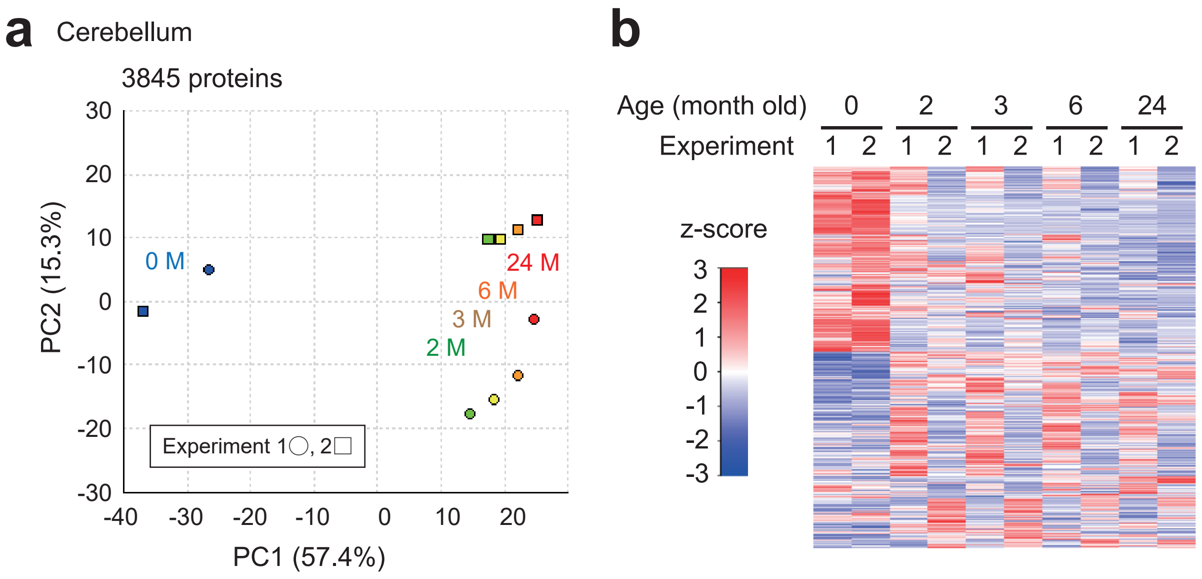


**Supplementary Fig. 13. PSD proteome analysis of common marmoset cerebellum**

PSD samples prepared from 0-, 2-, 3-, 6-, and 24-month-old marmoset cerebellum were subjected to LC-MS/MS to perform label-free quantification. **(a)** PCA was performed using the relative abundance values of 3,846 proteins. **(b)** The heatmap shows the relative abundance of each protein. Source data are provided as a Source Data file.

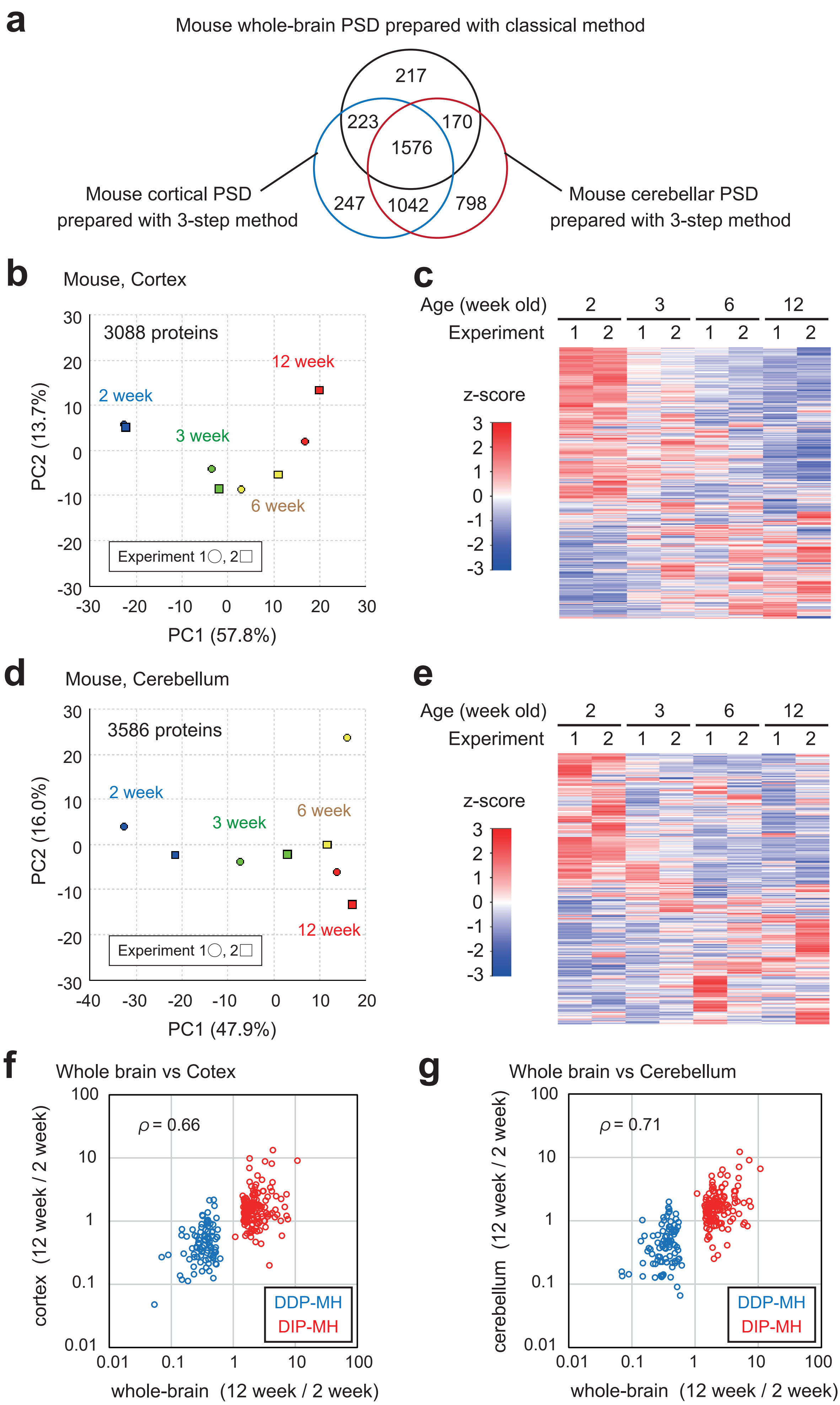
a
Mouse whole-brain PSD prepared with classical method
217
223
170
1576
247
1042
798
Mouse cortical PSD prepared with 3-step method
Mouse cerebellar PSD prepared with 3-step method
b
Mouse, Cortex
3088 proteins
2 week
3 week
6 week
12 week
Experiment 1○, 2□
PC1 (57.8%)
PC2 (13.7%)
c
Age (week old)
Experiment
z-score
d
Mouse, Cerebellum
3586 proteins
PC1 (47.9%)
PC2 (16.0%)
e
f
Whole brain vs Cotex
ρ = 0.66
DDP-MH
DIP-MH
cortex (12 week / 2 week)
whole-brain (12 week / 2 week)
g
Whole brain vs Cerebellum
ρ = 0.71
cerebellum (12 week / 2 week)

**Supplementary Fig. 14. Proteome analysis of mouse PSD using a 3-step method**

**(a)** Venn diagram illustrating the overlap of the analyzed proteins in the PSD of whole-brain prepared with the classical method or PSD of cortex or cerebellum prepared with the 3-step method. **(b-e)** PSD samples prepared from 2-, 3-, 6-, and 12-week-old mouse cortex (b and c) or cerebellum (d and e) were subjected to quantitative proteome analysis. Principal component analysis (PCA) was performed using the relative abundance values of 3,088 or 3,586 proteins (b and d). The heatmap shows the relative abundance of each protein (c and e). **(f and g)** Log10-fold change of the abundance of proteins in DDP-MH and DIP-MH groups in the developmental mouse cortex (f) or cerebellum (g) was plotted against that of the whole brain (12-week-old vs. 2-week-old). Source data are provided as a Source Data file.

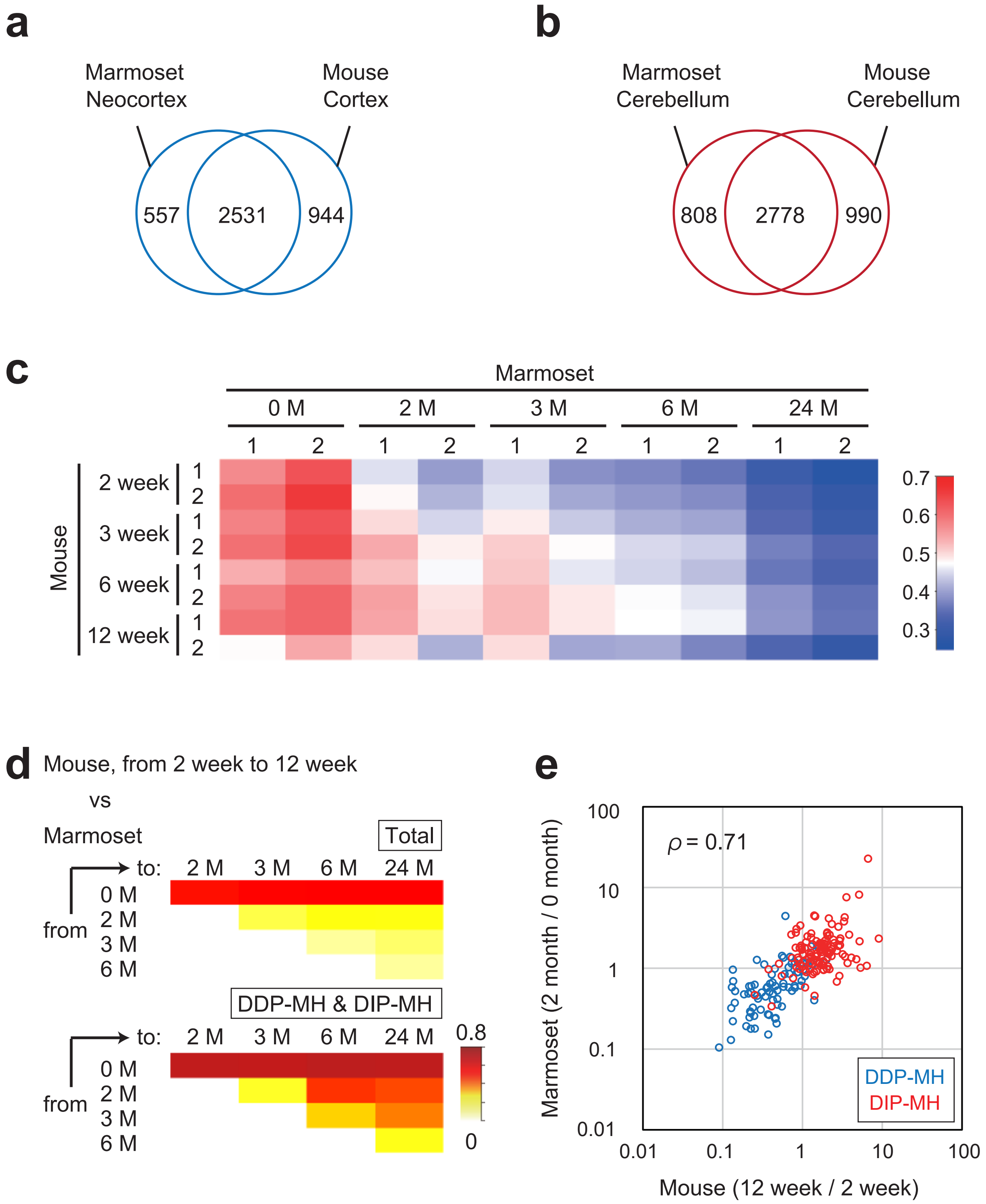


**Supplementary Fig. 15. Developmental alteration of PSD proteome in marmoset brain**

**(a and b)** Venn diagram illustrating the overlap of the analyzed proteins in the mouse and marmoset.Overlap of cortex (a) and cerebellum (b) are described, respectively. **(c)** Spearman's rank correlation coefficient of relative PSD protein abundance changes in mouse and marmoset cerebellum. For mouse data, the proteome of PSD prepared with the 3-step method was referred to. **(d)** Log10 fold change of the abundance of proteins in DDP-MH and DIP-MH groups in the developmental mouse cortex (12-week-old vs. 2-week-old) was plotted against that of marmoset neocortex at neonatal period (2-month-old vs 0-month-old). Source data are provided as a Source Data file.

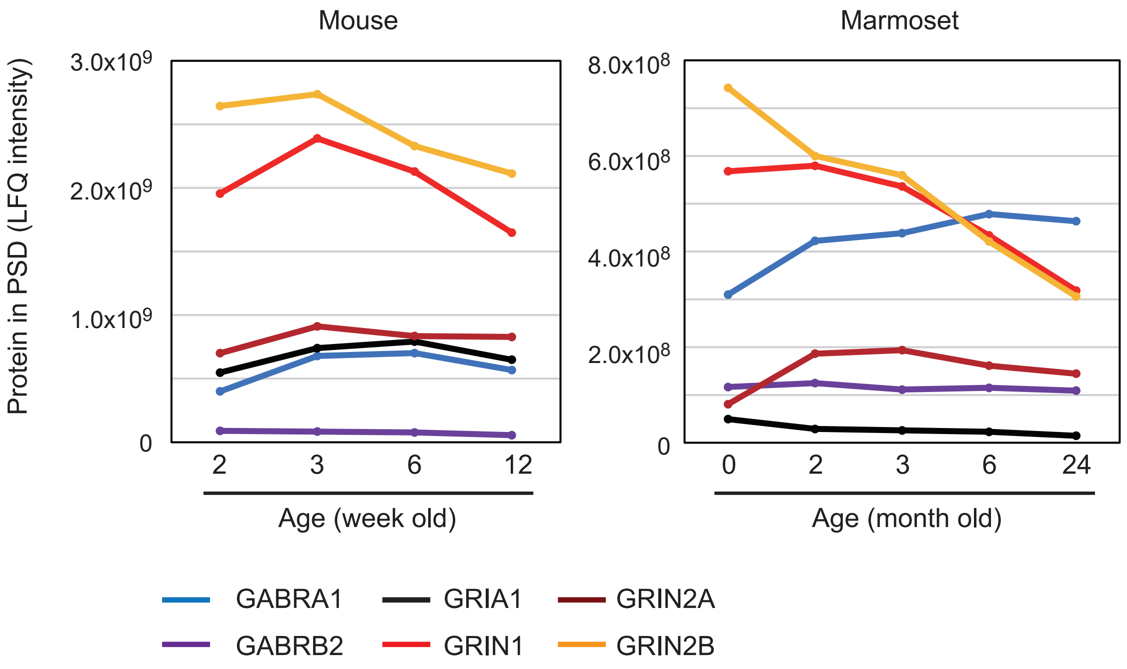


**Supplementary Fig. 16. Developmental trajectory of GABA receptor and glutamate receptor in mouse and marmoset**
Average abundance of GABA receptor subunits (GABRA1, GABRB2), NMDA receptor subunits (GRIN1, GRIN2A, and GRIN2B), and AMPA receptor subunit (GRIA1) is plotted referring Supplementary Data 6 and 8. Source data are provided as a Source Data file.

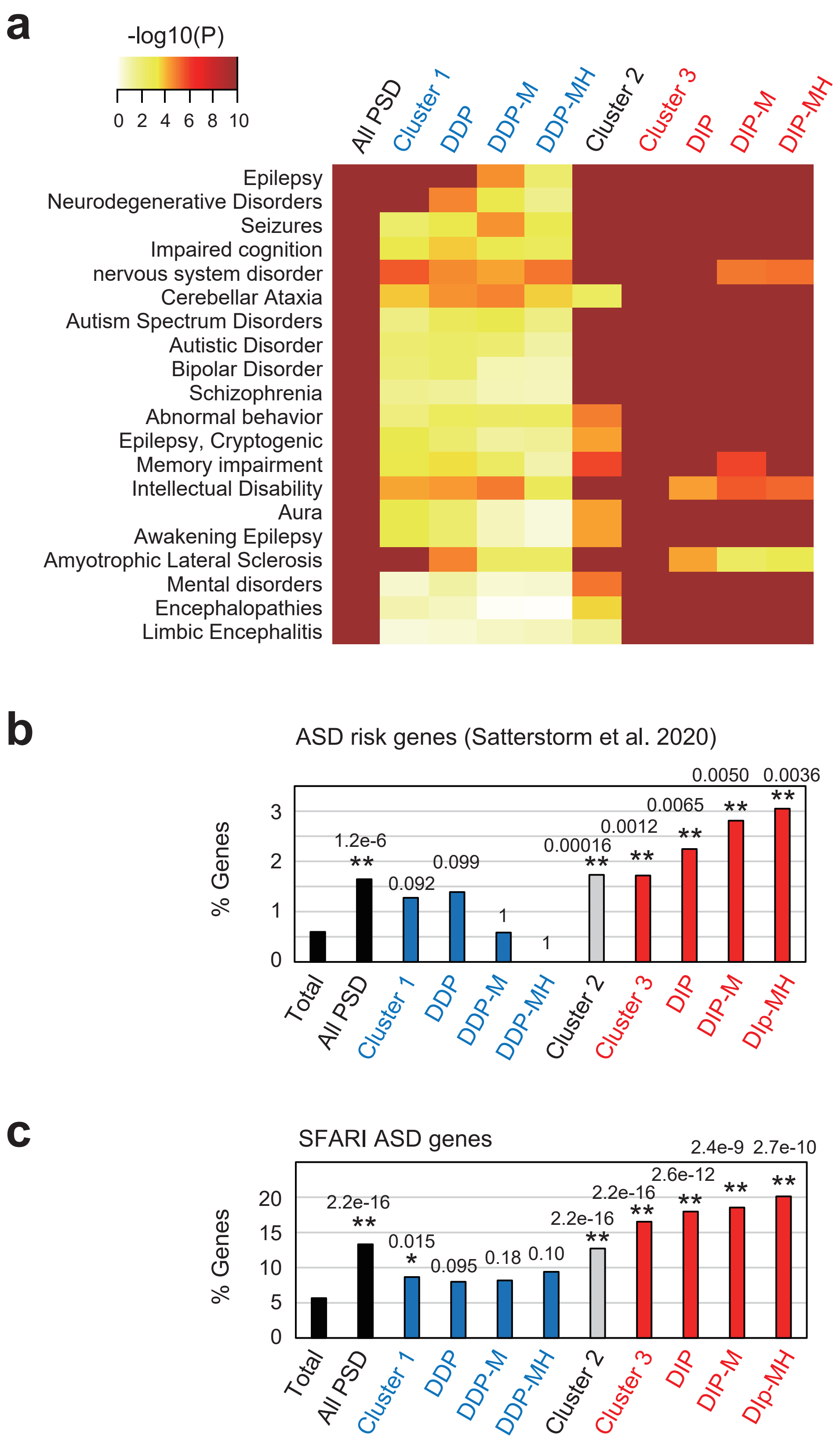


**Supplementary Fig. 17. Enrichment of genes related to neuropsychiatric disorders in genes encoding proteins on PSD** **(a)** Heatmap of diseases significantly enriched in PSD protein clusters. Disease enrichment analysis was performed using ToppCluster. **(b)** Enrichment of proteins encoded by ASD risk genes (Ref 9) **(c)** Enrichment of proteins encoded by SFARI ASD genes in PSD protein clusters. Asterisk indicates significant enrichment (*P < 0.05, **P < 0.01, Two-sided Fisher's exact test). Source data are provided as a Source Data file.

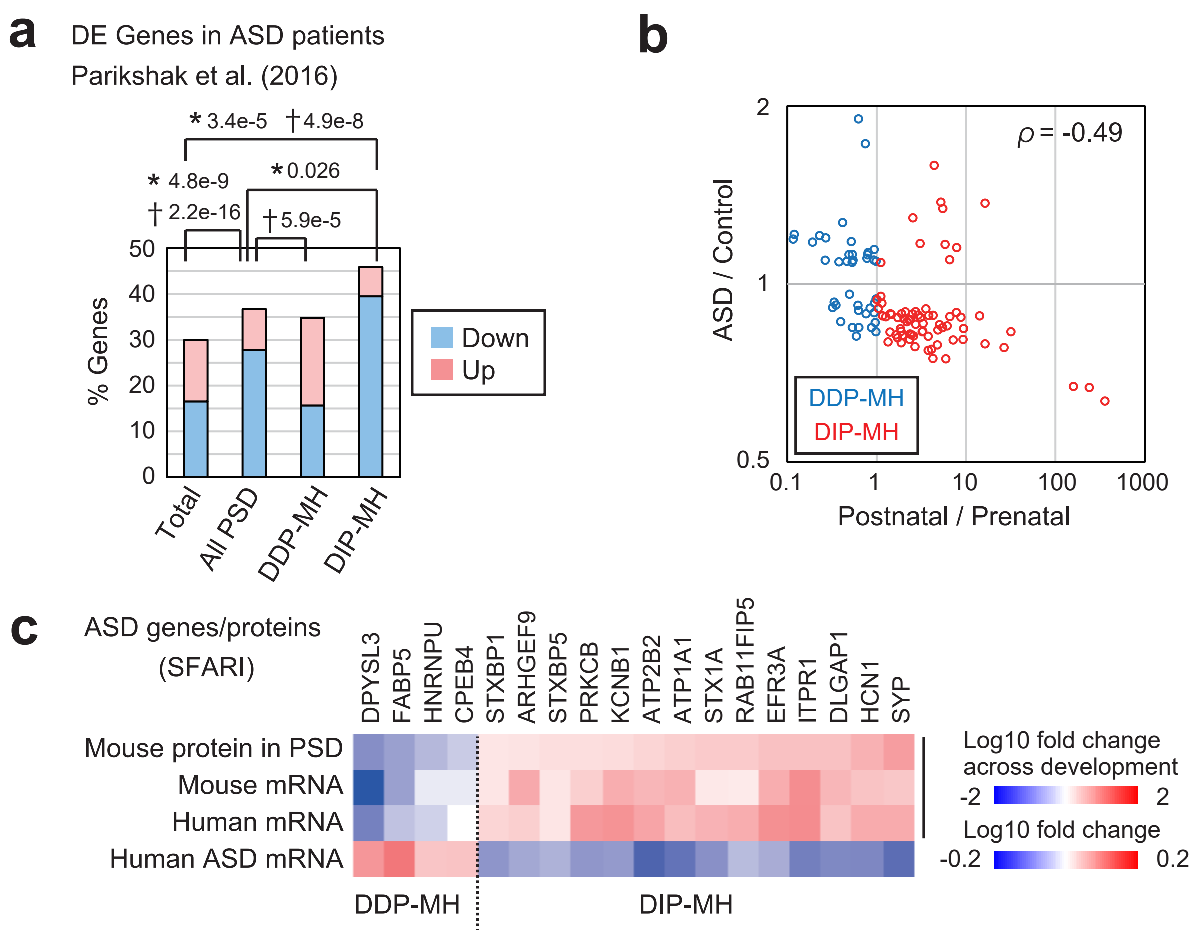


**Supplementary Fig. 18. Gene expression pattern related to PSD in individuals with ASD**

**(a)** The percentage of DE (upregulated and downregulated) genes in the cortical region of individuals with ASD (P < 0.05) (Ref 10). Gene set enrichment analysis was performed using two-sided Fisher's exact test. Asterisk and dagger indicate significant enrichment (P < 0.01) of DE genes and up- or down-regulated genes, respectively. **(b)** Log10 fold change of mRNA abundance in the developmental human cortex (postnatal vs. prenatal) (Ref 6) was plotted against that of individuals with ASD (ASD vs. control) (Ref 10). Red dotted lines indicate zero. **(c)** SFARI ASD genes showed a negative correlation: DDP-MH genes/proteins were increased in ASD, whereas DIP-MH genes/proteins were decreased in ASD. Heatmap describes the relative abundance of protein or mRNA; Mouse protein: protein abundance (P12w / P2w) (Table S2), Mouse mRNA: mRNA abundance (P180 / P4) (Ref 3), Human mRNA: mRNA abundance (Postnatal / Prenatal), Human ASD mRNA (ASD / Control) (Ref 10). Source data are provided as a Source Data file.

**Supplementary References**


1. Carlin, R. K., Grab, D. J., Cohen, R. S. & Siekevitz, P. Isolation and characterization of postsynaptic densities from various brain regions: Enrichment of different types of postsynaptic densities. *J. Cell Biol.* **86**, 831–843 (1980).
2. Weyn-Vanhentenryck, S. M. *et al.* Precise temporal regulation of alternative splicing during neural development. *Nat. Commun.* **9**, (2018).
3. Fertuzinhos, S. *et al.* Laminar and temporal expression dynamics of coding and noncoding RNAs in the mouse neocortex. *Cell Rep.* **6**, 938–950 (2014).
4. Kutzleb, C., Petrasch-Parwez, E. & Kilimann, M. W. Cellular and subcellular localization of paralemmin-1, a protein involved in cell shape control, in the rat brain, adrenal gland and kidney. *Histochem. Cell Biol.* **127**, 13–30 (2007).
5. Zhu, Y. *et al.* Spatiotemporal transcriptomic divergence across human and macaque brain development. *Science (80-. ).* **362**, (2018).
6. Li, M. *et al.* Integrative functional genomic analysis of human brain development and neuropsychiatric risks. *Science (80-. ).* **362**, (2018).
7. Oki, S. *et al.* Ch IP -Atlas: a data-mining suite powered by full integration of public Ch IP -seq data . *EMBO Rep.* **19**, 1–10 (2018).
8. Bayés, À. *et al.* Human post-mortem synapse proteome integrity screening for proteomic studies of postsynaptic complexes. *Mol. Brain* **7**, 1–11 (2014).
9. Satterstrom, F. K. *et al.* Large-Scale Exome Sequencing Study Implicates Both Developmental and Functional Changes in the Neurobiology of Autism. *Cell* **180**, 568-584.e23 (2020).
10. Parikshak, N. N. *et al.* Genome-wide changes in lncRNA, splicing, and regional gene expression patterns in autism. *Nature* **540**, 423–427 (2016).
