# Supplementary material for: Remodeling of the postsynaptic proteome in male mice and marmosets during synapse development
Source: Nat Commun. 2024 Mar 28;15:2496. doi: 10.1038/s41467-024-46529-9 (PMC10979008; doi:10.1038/s41467-024-46529-9)
Supplement: Supplementary file 3 — Description of Additional Supplementary Files [file 41467_2024_46529_MOESM3_ESM.pdf]

Description of Additional Supplementary Files

File Name: Supplementary Data 1 - 9
Description:

Supplementary Data 1: Full list of proteins detected in PSD in postnatal mouse brains.

Supplementary Data 2: LC-MS/MS quantitation and comparison of 2,186 PSD proteins in postnatal mouse brains.

Supplementary Data 3: Full results of canonical pathway analysis using IPA.

Supplementary Data 4: List of differentially expressed PSD proteins that have been reported to regulate the number, shape, or function of synapses.

Supplementary Data 5: LC-MS/MS quantification and comparison of 1,960 PSD proteins in seven brain regions in adult marmoset.

Supplementary Data 6: LC-MS/MS quantification and comparison of 3,535 PSD proteins in the neocortex of marmoset at postnatal development.

Supplementary Data 7: LC-MS/MS quantification and comparison of 3,846 PSD proteins in the cerebellum of marmoset at postnatal development.

Supplementary Data 8: LC-MS/MS quantitation and comparison of 3,088 PSD proteins in postnatal mouse cortex.

Supplementary Data 9: LC-MS/MS quantitation and comparison of 3,586 PSD proteins in postnatal mouse cerebellum.
