# Supplementary material for: Remodeling of the postsynaptic proteome in male mice and marmosets during synapse development
Source: Nat Commun. 2024 Mar 28;15:2496. doi: 10.1038/s41467-024-46529-9 (PMC10979008; doi:10.1038/s41467-024-46529-9)
Supplement: Supplementary file 5 — Reporting Summary [file 41467_2024_46529_MOESM5_ESM.pdf]

nature portfolio

Corresponding author(s): Toru Takumi

Last updated by author(s): Jan 18, 2024

Reporting Summary

Nature Portfolio wishes to improve the reproducibility of the work that we publish. This form provides structure for consistency and transparency in reporting. For further information on Nature Portfolio policies, see our Editorial Policies and the Editorial Policy Checklist.

Statistics

For all statistical analyses, confirm that the following items are present in the figure legend, table legend, main text, or Methods section.

n/a	Confirmed	
☐	☒	The exact sample size (*n*) for each experimental group/condition, given as a discrete number and unit of measurement
☐	☒	A statement on whether measurements were taken from distinct samples or whether the same sample was measured repeatedly
☐	☒	The statistical test(s) used AND whether they are one- or two-sided<br>*Only common tests should be described solely by name; describe more complex techniques in the Methods section.*
☐	☒	A description of all covariates tested
☐	☒	A description of any assumptions or corrections, such as tests of normality and adjustment for multiple comparisons
☐	☒	A full description of the statistical parameters including central tendency (e.g. means) or other basic estimates (e.g. regression coefficient) AND variation (e.g. standard deviation) or associated estimates of uncertainty (e.g. confidence intervals)
☐	☒	For null hypothesis testing, the test statistic (e.g. *F*, *t*, *r*) with confidence intervals, effect sizes, degrees of freedom and *P* value noted<br>*Give P values as exact values whenever suitable.*
☒	☐	For Bayesian analysis, information on the choice of priors and Markov chain Monte Carlo settings
☐	☒	For hierarchical and complex designs, identification of the appropriate level for tests and full reporting of outcomes
☐	☒	Estimates of effect sizes (e.g. Cohen's *d*, Pearson's *r*), indicating how they were calculated

Our web collection on statistics for biologists contains articles on many of the points above.

Software and code

Policy information about availability of computer code

Data collection	The MS and MS/MS data were searched against the NCBI-nr using Proteome Discoverer version 2.2 (Thermo Fisher Scientific) with the MASCOT search engine software version 2.6 (Matrix Science).
Data analysis	Statistical analyses were performed using R software (version 3.4.4 and 4.1.0). Heatmaps were generated in using the heatmap.2 function in the gplots package. For k-means clustering, the number of clusters was assessed using the NbClust package. One-way analysis of variance (ANOVA) was performed using the oneway.test function. Correction of p-value with Benjamini-Hochberg method was performed using the p.adjust function. The correlation coefficient was calculated using the cor.test function. Fisher's exact test was performed using the fisher.test function. The detailed R codes used in this study are available on GitHub (https://github.com/Takeshi-Kaizuka/Kaizuka_Proteomics_2024).<br>Enrichment of proteins reported to be expressed on PSD was evaluated using DAVID version 6.8. GO analysis and pathway analysis was performed using Metascape and SynGO. Enrichment of transcription factor binding on the genes was analyzed using ChIP-Atlas. Enrichment of disease-related genes and transcription binding sites were analyzed using ToppCluster. Canonical pathway analysis and network analysis were performed using Ingenuity Pathway Analysis (QIAGEN).

For manuscripts utilizing custom algorithms or software that are central to the research but not yet described in published literature, software must be made available to editors and reviewers. We strongly encourage code deposition in a community repository (e.g. GitHub). See the Nature Portfolio guidelines for submitting code & software for further information.

Data

Policy information about availability of data

All manuscripts must include a data availability statement. This statement should provide the following information, where applicable:

- Accession codes, unique identifiers, or web links for publicly available datasets
- A description of any restrictions on data availability
- For clinical datasets or third party data, please ensure that the statement adheres to our policy

The raw data of proteomics have been deposited in EBI-PRIDE (accession code: PXD048549). Source data are provided with this paper.
Previously reported proteome datasets of PSD fraction (Refs 34–48) were described in these articles. The full list of proteins is summarized and described in our preprint article (Ref 91). For transcriptome of developing mouse brain, we used two datasets; Dataset 1 (Ref 30) and Dataset 2 (Ref 23). Dataset 1 was downloaded from the NCBI website (https://www.ncbi.nlm.nih.gov//sra/?term=SRP055008) and converted to expression level (TPM) with RSEM (Ref 92). Transcriptome datasets and histone acetylation datasets of the developing human brain (Ref 26) and macaque brain (Ref 27) were downloaded from the PsychENCODE website (human transcriptome: http://development.psychencode.org/) (macaque transcriptome: http://evolution.psychencode.org/) (human histone acetylation: http://development.psychencode.org/#). For transcriptome of human ASD patient brain, data described in Ref 75 was referred. A list of SFARI ASD genes (released on 01-11-2022) was downloaded from the SFARI website (https://gene.sfari.org/database/human-gene/).

Research involving human participants, their data, or biological material

Policy information about studies with human participants or human data. See also policy information about sex, gender (identity/presentation), and sexual orientation and race, ethnicity and racism.

Reporting on sex and gender	N/A
Reporting on race, ethnicity, or other socially relevant groupings	N/A
Population characteristics	N/A
Recruitment	N/A
Ethics oversight	N/A

Note that full information on the approval of the study protocol must also be provided in the manuscript.

Field-specific reporting

Please select the one below that is the best fit for your research. If you are not sure, read the appropriate sections before making your selection.

[x] Life sciences [ ] Behavioural & social sciences [ ] Ecological, evolutionary & environmental sciences

For a reference copy of the document with all sections, see nature.com/documents/nr-reporting-summary-flat.pdf

Life sciences study design

All studies must disclose on these points even when the disclosure is negative.

Sample size	We didn't perform sample-size calculation. As for mouse samples, we chose n=4 to examine statistical significance of the results, considering the maximum number of samples that we can manipulate at once. As for marmoset samples, we chose n=2 doe to the limitation of sample availability
Data exclusions	In the proteome analyses, we extracted major proteins according to the three criteria and eliminated the other proteins from the following analysis unless otherwise stated; (1) at least two unique peptides were identified, (2) quantified in all datasets, and (3) coefficient of variation < 100. In the case when multiple proteins are encoded by a single gene, we selected a single protein encoded by a single gene whose signal intensity is highest.
Replication	We performed the experiments taking biological replicates as described in the manuscript to confirm the reproducibility of the results. There is no more successful or unsuccessful replication data that are not described in the manuscript.
Randomization	No randomization was performed in this study. Considering the technical variation of sample preparation, we grouped the subsets of individual age (e.g. 2, 3, 6, and 12-week-old) and repeated preparation of indicated number of samples.
Blinding	In this study, we didn't apply blinding of the samples for following reasons.<br>(1) Because different number of mouse brains was needed to prepare single PSD sample from different age (see Methods), we couldn't apply blinding. (2) There is some differences in appearance of marmoset brain at different age, which makes the blinding difficult.<br>The sample preparation and mass spectrometry was performed by different researchers and the latter researcher didn't have any biological hypothesis or bias. So, the lack of blinding doesn't affect the results of this study.

Reporting for specific materials, systems and methods

We require information from authors about some types of materials, experimental systems and methods used in many studies. Here, indicate whether each material, system or method listed is relevant to your study. If you are not sure if a list item applies to your research, read the appropriate section before selecting a response.

Materials & experimental systems

n/a	Involved in the study
☐	☒ Antibodies
☒	☐ Eukaryotic cell lines
☒	☐ Palaeontology and archaeology
☐	☒ Animals and other organisms
☒	☐ Clinical data
☒	☐ Dual use research of concern
☒	☐ Plants

Methods

n/a	Involved in the study
☒	☐ ChIP-seq
☒	☐ Flow cytometry
☒	☐ MRI-based neuroimaging

Antibodies

Antibodies used	Primary antibodies:<br>Rabbit anti-PSD-95 antibody (ab18258, Abcam)<br>Rabbit anti-Synaptophysin antibody (#4329, Cell Signaling Technology)<br>Rabbit anti-p-cofilin Ser3 (Cell Signaling #3313)<br>Rabbit anti-cofilin (Cell Signaling #5175)<br>Rabbit anti-Paralemmin-1 antisera (Ref 85)<br>Rabbit anti-PLCβ1 antibody (Ref 86)<br>Mouse anti-β-Actin antibody (A1978, SIGMA)<br>All primary antibodies were used in 1:1000 dilution.<br><br>Secondary antibodies:<br>Alexa Fluor 680- conjugated anti-rabbit IgG antibody (A-21076, Thermo Fisher Scientific) 1:5000<br>IRDye800CW-conjugated anti-mouse IgG antibody (610-131-121, Rockland Immunochemicals) 1:5000<br>Peroxidase anti-rabbit IgG antibody (111-035-003, Jackson Immuno Research Laboratories, Inc.) 1:10000<br>Peroxidase anti-mouse IgG antibody (NA9310, Amersham) 1:10000
Validation	The validation of the primary antibodies are as follows.<br><br>Rabbit anti-PSD-95 antibody: major band detection at expected molecular weight in immunoblotting<br>Rabbit anti-Synaptophysin antibody: major band detection at expected molecular weight in immunoblotting<br>Rabbit anti-p-cofilin Ser3: major band detection at expected molecular weight in immunoblotting and loss of the band detection in brain sample incubated for a while without phosphatase inhibitor<br>Rabbit anti-cofilin: major band detection at expected molecular weight in immunoblotting<br>Rabbit anti-Paralemmin-1 antisera: See Ref 85<br>Rabbit anti-PLCβ1 antibody: See Ref 86<br>Mouse anti-β-Actin antibody: major band detection at expected molecular weight in immunoblotting

Animals and other research organisms

Policy information about studies involving animals; ARRIVE guidelines recommended for reporting animal research, and Sex and Gender in Research

Laboratory animals	2, 3, 6, and 12-week-old mice were purchased from Japan SLC Inc. (Shizuoka, Japan). The housing conditions for the mice are as follows; Temperature: 23–25°C, Humidity: 45–65%, Light-Dark cycle: Light (8:00–20:00) Dark (20:00–8:00). We also used 0, 2, 3, 6, and 24-month-old common marmosets (See Figure S12a and Methods for the detail).
Wild animals	N/A
Reporting on sex	In this study, we used male animals. This is mainly because our study include relationship between PSD composition and autism spectrum disorder (ASD). For ASD study, typically only male animals are used because they show behavioral phenotypes well.
Field-collected samples	N/A
Ethics oversight	The animal experiments were approved by the Animal Research Committee in RIKEN and Kobe University Institutional Animal Care and Use Committee.

Note that full information on the approval of the study protocol must also be provided in the manuscript.

Plants

Seed stocks	N/A
Novel plant genotypes	N/A
Authentication	N/A
